# Obsessive-Compulsive Symptoms in an Adolescent with Intellectual Disability

**DOI:** 10.1155/2022/4943485

**Published:** 2022-03-22

**Authors:** Zainab Al-Abdullah, Faisal A. Nawaz, Hawk M. Kair, Meshal A. Sultan

**Affiliations:** ^1^College of Medicine, Mohammed Bin Rashid University of Medicine and Health Sciences, Dubai, UAE; ^2^Mental Health Centre of Excellence, Al Jalila Children's Specialty Hospital, Dubai, UAE

## Abstract

Individuals with intellectual disability (ID) experience various psychiatric comorbidities including anxiety, depression, and obsessive-compulsive disorder (OCD) in a rate that is equivalent or higher than individuals without ID. Unfortunately, these cooccurring conditions are often missed during the evaluations due to various reasons, including their atypical presentation. In this case report, we present the clinical symptoms of an adolescent with mild ID who presented with irritability and was diagnosed with OCD following a comprehensive assessment. The treatment course is also summarized as well as the positive outcome to selective serotonin reuptake inhibitor (SSRI) medication. In this report, we discuss potential factors that increase the rate of psychiatric comorbidities including OCD in individuals with ID. Furthermore, in the context of limited research in this area, we recommend additional studies in order to build a detailed understanding of the clinical presentation of psychiatric cooccurring disorders in individuals with ID with the goal of enhancing assessment tools in the future.

## 1. Introduction (Background)

Obsessive-compulsive disorder (OCD) presents as repetitive patterns of distressing thoughts, images, or impulses defined as obsessions. Common obsessions include violence, sexual, socially taboo topics, microbial contamination, and paranoia [1]. As such, patients respond to their obsessions with additional repetitive patterns of behaviors or mental acts called compulsions [1]. The amalgamation of obsessions and compulsions tends to debilitate a patient's daily life [1], despite having adequate insight regarding these patterns. A survey done in 1997 showed an OCD prevalence of 0.7% among those aged 18 to 65 [2]. The results of a survey from 2001 found those aged 5-15 at an overall OCD prevalence of 0.25% [3]. Furthermore, it revealed that OCD rarely presents in young children; however, the onset of symptoms is generally during adolescence [3, 4]. Diagnosing is further challenged by the difficulty of assessing obsessions with varying genetic risk factors and pediatric presentations. [1, 5, 6]. Psychiatric comorbidities reported in individuals with OCD include mood disorders, tic disorders, attention-deficit/hyperactivity disorder (ADHD), and developmental abnormalities [7, 8].

Intellectual disability (ID) is characterized by limitation in intellectual functioning and adaptive behavior during the early stages of psychological development. It is prevalent in about 1% of the general population [9] and carries a known association with mental illness [9–11]. Additionally, 3.5% of those aged between 11 and 62 with ID experience OCD symptoms and are likely to have a higher intelligence quotient (IQ) compared to other individuals with ID [6]. Another study found 0.8% of those aged between 3 and 18 with ID were also diagnosed with OCD, with a predilection towards the milder form of ID [12]. The pediatric OCD prevalence with or without ID was the same at 0.2% [10]. Unfortunately, it is difficult to diagnose mental disorders in patients with ID due to already impaired mental, social, and communication functions, making it challenging to observe changes in behavior associated with the onset of mental illness [13].

Remission of OCD is more likely before adulthood [7]. However, there is a 40% persistence of pediatric OCD into adulthood [8]. A worse prognosis may be associated with increased severity of initial presentation, delayed diagnosis and treatment, family history of anxiety and tic disorders, early age of onset, and existing mood or psychiatric comorbidities [7, 14]. Chronic OCD can be prevented with appropriate diagnosis and treatment, allowing patients to lead a relatively normal life regardless of chronicity [8]. Cognitive-behavioral therapy (CBT) is an effective first-line treatment of pediatric OCD, although it can be unadhered and complicated by psychiatric comorbidities, unsupportive environments, cognitive impairment (including ID), severity of initial presentation, and reduced accessibility to CBT compared to pharmacological treatment [8, 15].

The aim of this case report is to emphasize on the importance of carefully evaluating behavioral changes in individuals with ID, since psychiatric symptoms in this population may have an atypical presentation. We describe the clinical presentation of an adolescent with mild ID who developed OCD. We will refer to the adolescent in this report using “patient.”

## 2. Case Presentation

This patient is a 17-year-old adolescent of Arab origin. She is a grade 12 student in a regular class and has an individualized education plan. The patient lives with her mother, stepfather, and one younger brother.

When the patient was in grade 1, she was noticed to be slow in learning new concepts. She had difficulties working independently. Furthermore, she had challenges in keeping up with curriculum demands in all subjects. Her academic challenges persisted requiring her to receive, and she has been requiring significant support. Additionally, the patient has been experiencing high levels of anxiety since early childhood. As a result, she is described to be strongly attached to her mother.

At the age of 16 years, the patient was evaluated by a neuropsychologist. The Wechsler Intelligence Scale for Children-Fifth Edition (WISC-V) [16] revealed a full-scale intelligence quotient (FSIQ) score of 60. The Adaptive Behavior Composite (ABC) [17] revealed a score for daily living skill domain at the 1st percentile, communication domain at the 3rd percentile, and socialization domain at the 1st percentile. The impression from the neuropsychological evaluation was that the patient's presentation was in keeping with mild ID. This was based on FSIQ score being 60 (i.e., in the range between 50 and 69) and associated impairment in adaptive functioning as reflected by the ABC scores.

The patient was assessed at the outpatient clinic by a consultant child and adolescent psychiatrist. She presented with her mother due to concerns related to getting easily irritated when around her family. This included screaming, spitting, pulling her hair, and slamming doors. This issue started around the age of 15 years and has been waxing and waning in severity. She has been noted to be more irritable over the first couple of weeks of the academic year. However, no specific stressors other than starting school were identified. Her irritability impacted her relationship with her parents and brother. During the psychiatric assessment, the patient revealed that she has been experiencing recurrent disturbing thoughts in relation to contamination, harm to others, and fears of sexual nature. Her mother has noted that she would go to the washroom for 20 to 30 times a day, spending more than two hours in total. The patient indicated that she frequently visited the washroom at school as well. She indicated that during mealtime with her family, she gets a disturbing recurring thought about saliva getting out from the mouth of the people surrounding her and entering to her mouth. This thought was associated with significant distress and excessive fear of contamination. Additionally, the patient tends to get nervous and irritable during these periods. This results in spending excessive time in the washroom to gargle. Additionally, she reported checking the security camera excessively during the day because of a disturbing recurrent image about leaving the house and harming strangers on the street. Moreover, she has been avoiding being close to her brother because of a disturbing thought about his private areas somehow touching her inappropriately. The patient indicated that there have not been any incidents of inappropriate nature between her and her brother; however, she has been lately feeling very anxious around their presence due to the aforementioned thoughts.

Regarding her developmental and medical history, the patient was born postterm at 42 weeks of gestation. She had a high birth weight of 4.3 kg. She has met all fine motor, gross motor, speech, language, and toilet training developmental milestones. However, she was noted to be slow in terms of social and emotional development and had difficulties in integrating with other children. She is generally healthy from a physical perspective and does not have any chronic medical disorders. In terms of family history, her biological father has excessive anxiety in social situations. However, he never sought an evaluation. She does not have other relatives who have a psychiatric disorder. On initial assessment, the physical examination did not reveal any abnormalities. Her vital signs were within normal. Results of the routine blood work including complete blood count, liver profile, renal function, and thyroid function test were within normal limits. Her blood level of vitamin D was low. She followed at the adolescent medicine clinic, and the vitamin D deficiency was treated with a course of ergocalciferol 50,000 units weekly.

The psychiatric evaluation was based on DSM-5 diagnostic criteria [18] and revealed a diagnosis of OCD. She was prescribed a Selective Serotonin Re-uptake Inhibitor (SSRI) medication, Sertraline 25 mg, in the morning. The medication dose was increased by 25 mg at 2-week intervals until reaching the dose of 100 mg in the morning. At one-month follow-up of taking Sertraline 100 mg, the patient reported a decrease in the intensity of obsessions in the abovementioned domains of contamination, harm, and sexual thoughts by 50%. At two-month follow-up, she indicated a further decline in intensity of obsessions, now by 60% compared to the time prior to initiating SSRI medication. The Sertraline medication dose was then increased to 150 mg in the morning. In about six weeks of maintaining the dose of 150 mg, the patient indicated that her OCD symptoms have not been present anymore. Her progress was determined based on remission of symptoms and improvement in functioning according to the DSM-5 criteria for OCD. Along with following up at the psychiatry clinic, she attended the behavioral psychology clinic every one to two months for support around behavior regulation. As her OCD symptoms improved, her mother described overall improvement in her ability to regulate her emotion and behavior. The remission of her OCD symptoms continued until her most recent follow-up visit, four months since stabilizing on sertraline 150 mg a day.

## 3. Discussion

An adolescent with mild ID who developed OCD was presented in this report. The initial concern that led to seeking a psychiatric consultation was in the context of irritability. Similarly, a previous case report has described the presentation of an adult with moderate ID and OCD who got prominently irritated and used to calm down following washing his hands repeatedly [19]. However, it is worth noting that the domains of obsessions and compulsions of the patient in the presented case were consistent with the common themes described in previous studies, which have not specifically evaluated individuals with ID [20–22].

Biopsychosocial factors have likely contributed to the etiology of the presenting symptoms in this case report. Evidence has suggested a shared genetic and environmental risk between ID and psychiatric disorders [23]. A comprehensive review by Pauls et al. stated that “neuroimaging studies have implicated the cortico–striato–thalamo–cortical circuit in the pathophysiology of the disorder, which is supported by the observation of specific neuropsychological impairments in patients with OCD, mainly in executive functions. Genetic studies indicate that genes affecting the serotonergic, dopaminergic and glutamatergic systems play a crucial part in the functioning of this circuit” [24].

A study evaluating the presence of psychiatric disorders in children and adolescents assessed 641 individuals with ID and 17774 individuals without ID [10]. It revealed a comorbidity rate of 36% for psychiatric disorders in children with ID compared to 8% in children without ID [10]. The study highlighted several psychosocial disadvantages as potential contributing factors to development of cooccurring psychiatric disorders. For instance, the rates of the following adversities were higher among children with ID: lone parent family, exposure to two or more negative life events, income poverty, primary caregiving with no educational qualifications, and maternal self-rated physical health less than “good” [10]. Furthermore, previous studies have highlighted that children's problems associated with ID have a marked negative impact on their parents' psychological well-being [25, 26].

Although the rate of psychiatric comorbidity among individuals with ID is high, there is a tendency to either underdiagnose or misdiagnose these disorders [27, 28]. One of the reasons that contribute to underdiagnosis includes the phenomenon of “diagnostic overshadowing,” for instance, assuming that emotional and behavioral disturbances as expected characteristics of ID [[27]; Borthwick-Duffy, 2014; [13, 29]]. This highlights the importance of careful and comprehensive evaluations of any changes that significantly impact the functioning of individuals with intellectual challenges.

## 4. Conclusion

The reported case demonstrates the presentation of obsessions and compulsions in an adolescent with ID. The symptomatology is similar to domains commonly described among individuals without ID; however, the initial reason for this encounter was in the context of irritability. Future research with larger sample sizes of adolescents with ID and OCD is highly recommended to understand the key characteristics of this population in more depth. This will likely contribute to enhancement of comprehensive evaluations to detect potential comorbidity.

## Data Availability

The data used to support the findings of this study are included within the article.
